# Non-alcoholic fatty liver disease is associated with a worse prognosis in patients with heart failure: A pool analysis

**DOI:** 10.3389/fendo.2023.1167608

**Published:** 2023-04-20

**Authors:** Min Qiu, Jiahuan Li, Shali Hao, Haoxiao Zheng, Xiaojie Zhang, Hailan Zhu, Xiaolin Zhu, Yunzhao Hu, Xiaoyan Cai, Yuli Huang

**Affiliations:** ^1^ Department of Cardiology, Shunde Hospital, Southern Medical University, Foshan, China; ^2^ Department of Scientific Research and Education, Shunde Hospital, Southern Medical University, Foshan, China; ^3^ Guangdong Provincial Key Laboratory of Cardiac Function and Microcirculation, Southern Medical University, Guangzhou, China; ^4^ The George Institute for Global Health, Faculty of Medicine, University of New South Wales, Sydney, NSW, Australia

**Keywords:** heart failure, non-alcoholic fatty liver disease, prognosis, mortality, meta-analysis

## Abstract

**Background and aims:**

Non-alcoholic fatty liver disease (NAFLD) is associated with a higher risk of heart failure (HF) than those without NAFLD. However, the prognostic impact of NAFLD in HF is still controversial. This meta-analysis aimed to explore the association between NAFLD and the risk of adverse outcomes in patients with HF.

**Methods:**

We searched multiple electronic databases (Embase, PubMed, and Google Scholar) for potentially related studies up to June 30, 2022. Cohort studies reported multivariable adjusted relative risks and 95% confidence intervals (CIs) of adverse outcomes in HF patients with NAFLD comparing those without NAFLD were included for analysis.

**Results:**

A total of six studies involving 12,374 patients with HF were included for analysis, with a median follow-up duration of 2.5 years. The pooled analysis showed that HF patients with NAFLD were associated with a significantly increased risk of major composite adverse outcomes (HR 1.61, 95% CI 1.25-2.07), all-cause mortality (HR 1.66, 95% CI 1.39-1.98), and HF hospitalization or re-hospitalization (HR 1.71, 95% CI 1.03-2.86).

**Conclusion:**

NAFLD is associated with a worse prognosis in patients with HF. Effective screening and treatment strategies are needed to improve the prognosis in HF patients with NAFLD.

## Introduction

It is increasingly recognized that heart failure (HF) is a serious, worldwide public health problem, afflicting more than 37.7 million individuals (1). Patients with HF suffer from frequent hospitalizations, reduced life quality as well as shortened life expectancy (2). Therefore, it is important to identify and manage risk factors associated with adverse outcomes in patients with HF in a timely manner.

Non-alcoholic fatty liver disease (NAFLD) is a potentially progressive liver disease that excludes alcohol and other obvious factors of hepatic impairment, including asymptomatic simple steatosis, relatively severe non-alcoholic steatohepatitis, cirrhosis and liver cancer (3, 4). It leads to increased mortality in general populations, and its causes of death are mostly cardiac and hepatic in origin (5–7). NAFLD is considered to be one of the most common chronic liver diseases, as its prevalence is up to 25-45% in the general population (8). Recent studies suggested that NAFLD was associated with an increased risk of HF in patients, which may be caused by insulin resistance, impaired glucolipid metabolism, chronic inflammatory activation, increased renin-angiotensin-aldosterone and sympathetic nervous system activity, or the altered expression of adipokines and gut microbiota (9, 10). Furthermore, the prevalence of NAFLD in HF patients is progressively increasing (11–13). However, it is still unclear whether coexisting NAFLD leads to a poorer prognosis in HF patients. Some observational studies have shown that NAFLD would increase the mortality and hospitalization rates in patients with HF (14–19), while others did not suggest such an association (20). Therefore, aiming to explore the associations between NAFLD and a worse prognosis in patients with confirmed HF, we conducted the meta-analysis.

## Methods

### Search strategy and selection criteria

We conducted this study according to the advice of the MOOSE (Meta-analysis of Observational Studies in Epidemiology) Group. Three electronic databases were searched, including Embase, PubMed, and Google Scholar for potential related studies up to June 30, 2022. Terms related to “non-alcoholic fatty liver disease” and “HF” were included in the search strategies. We limited the search in human studies without language restriction. **Supplementary File 1** provided a detailed search strategy for PubMed, and similar searching strategies for other electronic databases, but were adapted if necessary. The list of references in the included studies were also reviewed in detail to identify potential related studies.

The inclusion criteria were as follows: (a) the study population must be adults (>18 years of age); (b) the study type should be an observational study, such as a cohort study, nested case-control study, etc.; (c) the data of the article should be complete or can be calculated to provide the following data: adjusted clinical outcomes including mortality and risk of rehospitalization in HF patients with NAFLD, compared with those without NAFLD.

The exclusion criteria were as follows: (a) Duplicate articles; if multiple articles had data from the same cohort, only their latest published results were taken. (b) Studies that were unable to provide sufficient data to calculate adjusted hazard ratios (HRs), such as cross-sectional studies that lacked follow-up time or some studies that did not report adjusted risk outcomes.

### Data extraction and quality assessment

Two researchers (MQ and YH) independently searched electronic databases, carefully screened and reviewed articles that met the inclusion exclusion criteria, extracted information and data from the included studies and performed a literature quality assessment. Study information such as study design, ethnicity, participant numbers, sex, type of HF, the definition of NAFLD, average age, follow-up time and outcomes were extracted and recorded in a predefined format. If necessary, we would contact the author for additional data. The Newcastle-Ottawa Quality Assessment Scale was used to assess the quality of included study, which included 8 items over 3 domains: study population selection, comparability, and exposure/outcome, with a maximum of 9 points. The included studies were classified into three categories based on scores: good (≥7 points), fair (4–6 points) or poor (<4 points).

### Data analysis

We assessed whether coexisting NAFLD in HF patients was associated with a poorer prognosis compared with non-NAFLD patients. The primary outcome was all adverse outcomes including all cause/cardiac mortality, all cause/cardiac hospitalization or rehospitalization, combined cardiac mortality and hospitalization or rehospitalization. The secondary outcomes included the risks of all-cause/cardiac mortality, all-cause/cardiac hospitalization or rehospitalization. We extracted the data adjusting for the most confounding factors, if there were results of multiple adjusted models in the study. If NAFLD was defined by a quantitative metric (fibrosis-4 index [FIB-4], fatty liver index [FLI] or NAFLD fibrosis score [NFS]), the lowest quantile group was used as the referent group and the remaining quantile groups were combined as the exposure group for extraction of the HRs data. We used a random effects model to fit the results, and we used the inverse variance method to combine the HRs and corresponding standard errors. The results of the heterogeneity test are expressed by *I^2^* statistics, which indicates significant heterogeneity if *I^2^* is greater than 50%.

We performed the subgroup analyses of the primary outcome according to age, ethnicity, the definition of NAFLD, type of HF [based on course, ejection fraction (EF)], adjustment for confounders [B-type natriuretic peptide (BNP), body weight], sample sizes and follow-up time. We performed sensitivity analyses to explore the effect of random/fixed-effects models on the results, as well as manually removing one study at a time to test the stability of the results Funnel plots were used to assess qualitatively the publication bias of the included studies.

The Review Manager software (version 5.2 for Windows, The Cochrane Collaboration, Copenhagen, Denmark) was used in all data analyses. Two-sided *P* values <0.05 were considered statistically significant.

## Results

### Characteristics of included studies

**Figure 1:**
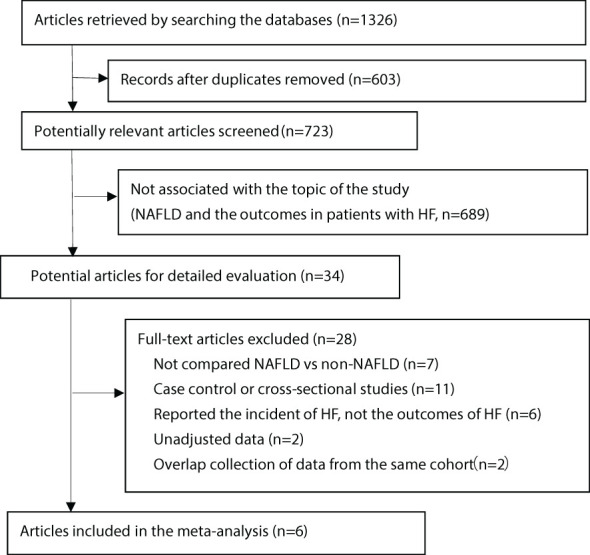
Flow of papers through screening and review. HF, heart failure; NAFLD, non-alcoholic fatty liver disease.

The initial search resulted in 1326 items. We screened the titles and abstracts, and then identified 34 articles that required further comprehensive review (**Figure 1**). However, two articles (Valbusa 2016 (18) and Valbusa 2017 (17)) reported the same outcome from the same cohort, we only included the most updated and comprehensive data (from Valbusa 2017 (17)) for analysis. Similar situation was also observed in the other two aticles (Yoshihisa 2018 (16) and Sato 2017 (21)), and we included data from Yoshihisa’s study for analysis (16). Finally, 6 observational studies were enrolled in the analysis, which included 12,374 participants with a median duration of 2.5 years. The key characteristics of the included studies are presented in **Table 1**. Three of the studies included in the analysis were from Asians and three of them were from Europe and Americas. There were two articles that included only acute HF, only one included chronic HF, while three without specifying the type of HF according to the course of the disease. All of the studies excluded people with known chronic liver disease except NAFLD.

**Table 1:** Characteristics of the included studies.

Study	Country	Study design	Sample size (male%)	Average age (years)	Type of HF	Definition of NAFLD	Follow-up (years)	Outcomes
Takahashi. 2017	Japan	Prospective cohort study	516 (58.7)	71.0 ± 12.8	CHF	NFS	1.3	Combination of cardiac mortality and rehospitalization
Valbusa. 2017 (17)	Italy	Prospective cohort study	212 (45.3)	82.0 ± 9.0	AHF	Ultrasonography	1.0	1. All-cause rehospitalization 2. Cardiac rehospitalization
Valbusa. 2018 (14)	Italy	Prospective cohort study	264 (51.1)	83.0 ± 9.0	AHF	Ultrasonography	1.0	All-cause mortality
Yoshihisa. 2018 (16)	Japan	Prospective cohort study	492 (49.8)	69.8	HFpEF	NFS	3.0	All-cause mortality
Park J. 2021 (19)	Korean	Prospective cohort study	7445 (NA)	52.4	Not specified	FLI	8.5	1. All-cause mortality 2. Cardiac mortality 3. Cardiac hospitalization
Peters. 2021 (20)	Six countries*	Post hoc trial	3445(48.5)	68.7	HFpEF	NFS, FIB-4	3.3	1. All-cause hospitalization 2. Cardiac hospitalization 3. Combination of cardiac mortality and hospitalization

HF, heart failure; AHF, acute heart failure; CHF, chronic heart failure; HFpEF, heart failure with preserved ejection fraction; NAFLD, non-alcoholic fatty liver disease; FIB-4, Fibrosis-4; NFS, non-alcoholic fatty liver disease fibrosis score; FLI, fatty liver index; NA, not available.

*United States, Canada, Brazil, Argentina, Russia, and Georgia

Two studies used ultrasonography to define NAFLD, 2 used NFS and 1 used FLI. One study used both FIB-4 and NFS to define NAFLD, we used NFS for analysis first, and then included FIB-4 in the sensitivity analysis (20). They were calculated using the following formulas:

FIB-4=[age (years) * aspartate aminotransferase (AST, U/L)]/[platelet (* 10^9^/L) *√ alanine aminotransferase (ALT, U/L)] (20, 21);

NFS=[−1.675 +.037 * age (years) +.094 * body mass index (BMI, kg/m^2^) + 1.13 * impaired fasting glucose or diabetes (yes=1, no=0) +.99 * AST/ALT ratio −.013 * platelet (* 109/L) −.66 * albumin (g/dL)] (15, 16, 20);

FLI=(e^.95 *log_e_ (triglyceride) +.139 * BMI +.718 * log_e_ (gamma-glutamyl transferase) + .053 *waist circumference (WC) − 15.745)^/(1 + e^.95 * log_e_ (triglyceride) + .139 *BMI +.718 × log_e_ (gamma-glutamyl transferase) + .053 *WC − 15.745^) * 100^19^.

The confounding factors adjusted for each study are summarized in **Supplementary File 2**. We found that almost all included studies met a good quality rating according to the scoring rules of the Newcastle-Ottawa Quality Assessment, with the exception of one study (**Supplementary File 3**).

### Association between NAFLD and adverse outcomes in HF patients

**Figure 2:**
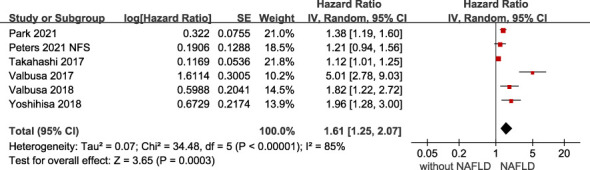
Association between NAFLD and the risk of primary adverse outcomes in patients with heart failure. CI, confidence interval; NAFLD, non-alcoholic fatty liver disease.

**Figure 3:**
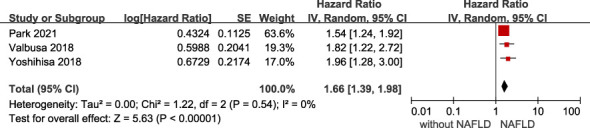
Association between NAFLD and the risk of all-cause/cardiac mortality in patients with heart failure. CI, confidence interval; NAFLD, non-alcoholic fatty liver disease.

**Figure 4:**
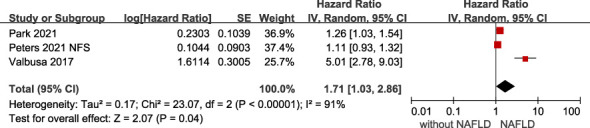
Association between NAFLD and the risk of all-cause/cardiac hospitalization or rehospitalization in patients with heart failure. CI, confidence interval; NAFLD, non-alcoholic fatty liver disease.

The association of NAFLD with the risk of adverse outcomes in patients with confirmed HF was reported in total 6 studies, but they showed significant heterogeneity among them (*I^2 =^* 85%, *p* <.001). Overall, we found a significantly increased risk of primary adverse outcomes (HR 1.61, 95% CI 1.25-2.07) when patients with HF coexisted with NAFLD (**Figure 2**). After visually inspecting the funnel plot, we identified no support for the presence of publication bias (**Supplementary File 4**). Furthermore, NAFLD was associated with a 66% increased risk of all-cause mortality (HR 1.66, 95% CI 1.39-1.98, *I^2 =^* 0%) in HF patients (**Figure 3**), and a 71% increased risk of HF hospitalization or re-hospitalization (HR 1.71, 95% CI 1.03-2.86, *I^2 =^* 91%) (**Figure 4**), compared with those without NAFLD.

### Subgroup analyses and sensitivity analyses

We performed subgroup analyses of the primary outcomes and presented the results in **Table 2**. The risk of primary adverse outcomes was all increased among subgroup analyses performed according to age, different ethnicities, definition of NAFLD, sample sizes, adjustment for BNP or body weight, and type of HF (based on course). Although the risk of the primary outcomes did not reach statistically significant in the subgroups with less than 3 years of follow-up (HR 2.07, 95% CI 0.97-4.40) or patients with heart failure with preserved ejection fraction (HFpEF) (HR 1.49, 95% CI 0.93-2.38), no significant heterogeneity was found among the subgroups comparisons (both *P* for interaction >0.10)

**Table 2:** Subgroup analyses of the association between NAFLD and primary adverse outcomes in patients with heart failure.

Subgroup	No. of comparisons	HR (95% CI)	P-value	P-for interaction
Average age (years)				0.11
≥80	2	2.95 [1.09, 7.94]	0.03	
<80	4	1.30 [1.09, 1.55]	0.003	
Ethnicity				0.23
Asians	3	1.35 [1.07, 1.69]	<0.01	
Non-Asians	3	2.13 [1.04, 4.35]	<0.04	
Definition of NAFLD				0.28
Ultrasonography	2	2.95 [1.09, 7.94]	0.03	
NFS	3	1.29 [1.01, 1.65]	0.04	
FLI	1	1.38 [1.19, 1.60]	<0.001	
Follow-up duration (years)				0.33
<3	3	2.07 [0.97, 4.40]	0.06	
≥3	3	1.40 [1.15, 1.70]	0.0007	
Sample sizes				0.16
<1000	4	2.02 [1.14, 3.56]	0.02	
≥1000	2	1.33 [1.17, 1.52]	<0.001	
Adjusted for BNP				0.16
Yes	4	2.02 [1.14, 3.56]	0.02	
No	2	1.33 [1.17, 1.52]	<0.001	
Type of HF (based on course)				0.11
AHF	2	2.95 [1.09, 7.94]	0.03	
Not AHF	4	1.30 [1.09, 1.55]	0.003	
Type of HF (based on EF)				0.66
HFpEF	2	1.49 [0.93, 2.38]	0.09	
Not specified	4	1.70 [1.21, 2.40]	0.002	
Adjusted for body weight				0.52
Yes	2	1.49 [1.17, 1.89]	0.001	
No	4	1.75 [1.13, 2.72]	0.01	

NAFLD, non-alcoholic fatty liver disease; HR, hazard ratio; CI, confidence interval; BNP, B-type natriuretic peptide; HF, heart failure; AHF, acute heart failure; CHF, chronic heart failure; HFpEF, heart failure with preserved ejection fraction; FIB-4, Fibrosis-4; NFS, non-alcoholic fatty liver disease fibrosis score; FLI, fatty liver index

The results of all our sensitivity analyses were consistent in that the association between NAFLD and risk of adverse outcomes was unchanged, regardless of whether the random/fixed-effects model was changed or the HR was recalculated by omitting a study at a time (**Supplementary File 5**). Furthermore, the study by Peters et al. (20) used both FIB-4 and NFS to define NAFLD, we used NFS for the main analysis first, and then included FIB-4 in the sensitivity analysis. The results were similar (the risk of primary adverse outcome in NAFLD: HR 1.27, 95% CI 1.18-1.38).

## Discussion

To our knowledge, our comprehensive meta-analysis is the first study to assess the association of NAFLD with worse prognosis in patients with HF. After adjusting for multiple cardiovascular risk factors, we found that coexisting NAFLD was associated with a higher risk of adverse outcomes (all cause mortality and re-hospitalization) in HF patients, compared with those without NAFLD.

NAFLD may contribute to the suboptimal prognosis of patients with HF through multiple mechanisms. Firstly, the existence of NAFLD is probably related to the severity of HF. Previous studies have reported that HF was associated with elevated levels of certain indicators that were commonly used to assess the severity of NAFLD, such as serum GGT and transaminase levels (22). Secondly, oxidative stress and inflammation, insulin resistance, impaired lipocalin, and increased visceral adiposity are common pathophysiologic mechanisms in NAFLD that can trigger functional and structural alterations in the heart. It may play an important role in disease progression in HF patients with NAFLD (23–26). Finally, NAFLD was associated with increased renin-angiotensin-aldosterone and sympathetic nervous system activity, as well as excessive deposition of extracellular matrix collagen fibers, which could impact cardiac remodeling and might be an important factor in the prognosis of patients with HF.

Based on our findings, there are some clinical recommendations that should be proposed in the management of HF. Considering the prevalence of NAFLD, screening for NAFLD in patients with HF may provide effective detection of the disease, timely interventions and reduce the risk of adverse outcomes. Periodic monitoring of the ultrasonic echocardiogram index and clinical biomarkers, e.g, N-Terminal Pro-B-Type Natriuretic Peptide in HF patients with NAFLD would be important for early detection of the worsening of HF clinical course. The high-risk patient of HF combined with NAFLD may require individualized treatment. Some studies showed beneficial effects on patients with NAFLD through changes in dietary behavior, and continuously losing weight contributes to the improvement of liver fibrosis (27, 28). These lifestyle modifications should be proposed as the cornerstone in HF patients with NAFLD. Furthermore, it is important for prospective intervention studies to ascertain whether potential therapy for NAFLD would contribute to positive prognosis in heart failure patients, for instance, by improving insulin resistance or reducing oxidative stress with sodium glucose co-transporter 2 (SGLT-2) inhibitors or other new therapeutic agents (29–31).

According to ejection fraction, heart failure can be divided into three categories: HFpEF, reduced heart failure (HFrEF), and mildly reduced heart failure (HFmrEF) (32). Their prognosis and risk factors may be different. Novel drugs have significantly improved the prognosis of HFrEF during the past decade. However, limited progress has been achieved in the treatment of HFpEF (33). Novel risk stratification and treatment are needed to improve the outcomes of HFpEF. Unfortunately, in our analysis, the association between NAFLD and the prognosis in HFpEF did not reach statistical significance, although no significant heterogeneity was found among the subgroups comparisons. This may be caused by limited number of studies available for analysis, and only 2 studies reported the outcomes associated with NAFLD in HFpEF. Therefore, the role of NAFLD in patients with HFpEF is still needed investigation.

This meta-analysis has several strengths. Firstly, in the analysis of primary adverse outcomes, there were significant heterogeneity observed among the included studies (*I^2 =^* 85%, *P*<.001). As shown in subgroup analyses, a number of factors such as NAFLD definition, follow-up duration, type of HF, and adjusted confounding factors could affect the magnitude of the association between NAFLAD and prognosis in HF, and thus may serve as sources of heterogeneity. However, other possible sources of heterogeneity (such as gender, and body mass index) could not be explored due to the lack of available data. Secondly, in most of the included studies, HF was only categorized according to ejection fraction or time course, while the specific etiology was unclear. It also remained unknown whether NAFLD played a different role in different types of HF. Thirdly, only a relatively small number of studies were available for pool analysis, and the median follow-up duration was relatively short to draw a solid conclusion. Further large sample cohort studies with long follow-up duration were needed to document the role of NAFLD in HF. Finally, the gold standard for NAFLD is histopathological examination of liver tissue. In the enrolled studies, the cut off values in NAFLD definition, such as NFS or FIB-4 have not been uniformed yet. It was also unclear whether patients with more advanced NAFLD based on histopathological examination (such as NASH and liver fibrosis) suggesting an increased risk of adverse prognosis.

## Conclusion

NAFLD is associated with an increased risk of adverse outcomes in patients with HF. Effective screening and treatment strategies for NAFLD should be considered to improve the prognosis in HF patients.

## Data availability statement

The original contributions presented in the study are included in the article/**Supplementary Material**. Further inquiries can be directed to the corresponding authors.

## Author contributions

Conception and design: MQ, XC, YLH. Data curation: MQ, XZhang, HZhu. Formal analysis: MQ, JL. Writing - original draft: MQ, YLH. Writing - review & editing: all authors. All authors contributed to the article and approved the submitted version.
