# Supplementary material for: Non-alcoholic fatty liver disease is associated with a worse prognosis in patients with heart failure: A pool analysis
Source: Front Endocrinol (Lausanne). 2023 Apr 20;14:1167608. doi: 10.3389/fendo.2023.1167608 (PMC10157242; doi:10.3389/fendo.2023.1167608)
Supplement: Supplementary file 1 [file DataSheet_1.doc]

**Supplementary file 1. Literature search strategy for Pubmed**

| **#1** | "Non-alcoholic Fatty Liver Disease "[Mesh] |
| --- | --- |
| **#2** | (((((((Non-alcoholic Fatty Liver Disease[Text Word]) OR Non-alcoholic steato-hepatitis[Text Word]) OR NAFLD[Text Word]) OR NASH[Text Word]) OR steatohepatitis[Text Word]) OR steatosis[Text Word]) OR fatty liver[Text Word]) OR Liver fat[Text Word] |
| **#3** | #1 OR #2 |
| **#4** | Heart failure [Mesh] |
| **#5** | ((((("Heart failure"[Text Word]) OR " Cardiac Failure "[Text Word]) OR " Myocardial Failure "[Text Word]) OR " Cardiac dysfunction"[Text Word] OR " Heart dysfunction "[Text Word]) OR " Myocardial dysfunction "[Text Word]) |
| **#6** | #4 OR #5 |
| **#7** | #3 AND #6 |
| **#8** | animals[MeSH Terms] |
| **#9** | humans[MeSH Terms] |
| **#10** | #8 NOT #9 |
| **#11** | #7 NOT #10 |

**Supplementary file 2. Confounders adjusted in the included studies**

| **Study** | **Confounder adjusted** | **Number of adjustment** |
| --- | --- | --- |
| Takahashi. 2017 | NYHA functional class, hemoglobin, eGFR, and log BNP. | 4 |
| Valbusa. 2017 | Age, sex, hospital ward, past history of HF, diabetes, CHD, obesity, eGFR, LV ejection fraction, NT-proBNP, serum sodium and GGT levels. | 12 |
| Valbusa. 2018 | Age, sex, past history of HF, diabetes, CHD, CKD, COPD and presence of pacemakers/ICDs, hospital ward, body weight, systolic blood pressure, LV-ejection fraction, use of ACE-I/ARBs, daily furosemide dosages, plasma albumin, NT-proBNP and GGT concentrations. | 16 |
| Yoshihisa. 2018 | Sex, Nyha class III or IV, ischaemic aetiology, atrial fibrillation, chronic kidney disease, anaemia, BNP, hyponatraemia, renin–angiotensin–aldosterone system inhibitors, β-blockers, and diuretics. | 11 |
| Park. 2021 | Age, sex, body weight, alcohol drinking, smoking, regular exercise, income status, hypertension, diabetes mellitus, dyslipidemia, and estimated glomerular fltration rate. | 11 |
| Peters. 2021 | Sex, race, NYHA class, smoking, systolic blood pressure, sodium, blood urea nitrogen, prior cardiovascular disease, previous hospitalization for heart failure, and use of spironolactone. | 10 |

eGFR: estimated glomerular filtration rate, GGT: gamma-glutamyltransferase, NYHA: New York Heart Association, BNP: brain natriuretic peptide, ACE: angiotensin-converting enzyme, ARB: angiotensin receptor blocker, CHD: coronary heart disease, CKD: chronic kidney disease,COPD: chronic obstructive pulmonary disease, ICD: implantable cardiac defibrillator

**Supplementary file 3. Quality Assessment of the included studies**

| **Study** | **Selection (stars awarded)** | **Comparability (stars awarded)** | **Outcome (stars awarded)** | **Quality (total stars awarded)** |
| --- | --- | --- | --- | --- |
| Takahashi. 2017 | 3 | 1 | 2 | Fair (6) |
| Valbusa. 2017 | 4 | 2 | 2 | Good (8) |
| Valbusa. 2018 | 4 | 2 | 1 | Good (7) |
| Yoshihisa. 2018 | 4 | 1 | 3 | Good (8) |
| Park. 2021 | 4 | 2 | 3 | Good (9) |
| Peters. 2021 | 4 | 1 | 2 | Good (7) |

**Supplementary file 4. Funnel plot for evaluation of publication bias: The risk of adverse outcomes associated with NAFLD in patients with heart failure**


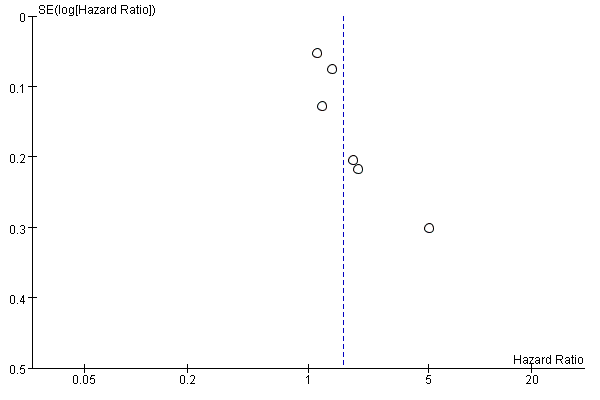


**Supplementary file 5. Sensitivity analyses of the association between NAFLD and primary adverse outcomes in patients with heart failure.**

| Item | HR (95% CI) | P-value |
| --- | --- | --- |
| fixed-effect | 1.27 [1.18, 1.38] | <0.001 |
| Different definition of NAFLD(FIB-4)^#^ | 1.55 [1.21, 1.99] | <0.001 |
| After omitting follow study |  |  |
| Park 2021 | 1.75 [1.20, 2.55] | <0.001 |
| Peters 2021 NFS | 1.75 [1.28, 2.38] | <0.001 |
| Takahashi 2017 | 1.81 [1.30, 2.53] | <0.001 |
| Valbusa 2017 | 1.36 [1.14, 1.63] | <0.001 |
| Valbusa 2018 | 1.57 [1.19, 2.08] | 0.001 |
| Yoshihisa 2018 | 1.55 [1.18, 2.03] | 0.001 |

NAFLD= non-alcoholic fatty liver disease; NFS= non-alcoholic fatty liver disease fibrosis score; FIB-4= Fibrosis-4;

# The study by Peters et al.^20^ used both FIB-4 and NFS to define NAFLD, we used NFS for the main analysis first, and then included FIB-4 in the sensitivity analysis.
